# 2-Amino-6-(quinoline-2-carboxamido)­pyridinium nitrate

**DOI:** 10.1107/S1600536812036562

**Published:** 2012-08-31

**Authors:** Phillipus C. W. Van der Berg, Hendrik G. Visser, Andreas Roodt

**Affiliations:** a Department of Chemistry, University of the Free State, PO Box 339, Bloemfontein 9300, South Africa

## Abstract

In the title salt, C_15_H_13_N_4_O^+^·NO_3_^−^, an extensive network of N—H⋯N, N—H⋯O and C—H⋯O hydrogen-bond inter­actions are observed throughout the structure. Further stabilization is obtained by π–π stacking inter­actions between inversion-related quinoline systems and inversion-related pyridine rings, with respective centroid–centroid distances of 3.5866 (6) and 3.3980 (6) Å.

## Related literature

For related radiopharmaceutical structures, see: Al-Dajani *et al.* (2010 ▶); Jain *et al.* (2004 ▶); Van der Berg *et al.* (2011 ▶).
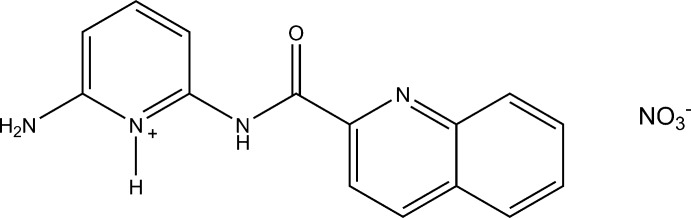


## Experimental

### 

#### Crystal data

C_15_H_13_N_4_O^+^·NO_3_^−^
*M* *_r_* = 327.30
Monoclinic, 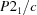

*a* = 8.183 (2) Å
*b* = 11.768 (3) Å
*c* = 14.979 (4) Å
β = 98.37 (1)°
*V* = 1427.1 (6) Å^3^
*Z* = 4
Mo *K*α radiation
μ = 0.12 mm^−1^
*T* = 100 K
0.49 × 0.41 × 0.31 mm

#### Data collection

Bruker X8 APEXII KappaCCD diffractometer
Absorption correction: multi-scan (*SADABS*; Bruker, 2008 ▶) *T*_min_ = 0.990, *T*_max_ = 0.994
26710 measured reflections
3555 independent reflections
3180 reflections with *I* > 2σ(*I*)
*R*_int_ = 0.023

#### Refinement

*R*[*F* ^2^ > 2σ(*F* ^2^)] = 0.035
*wR*(*F* ^2^) = 0.1
*S* = 1.04
3555 reflections
233 parameters
H atoms treated by a mixture of independent and constrained refinement
Δρ_max_ = 0.42 e Å^−3^
Δρ_min_ = −0.25 e Å^−3^

### 

Data collection: *APEX2* (Bruker, 2011 ▶); cell refinement: *SAINT-Plus* (Bruker, 2008 ▶); data reduction: *SAINT-Plus*; program(s) used to solve structure: *SHELXS97* (Sheldrick, 2008 ▶); program(s) used to refine structure: *SHELXL97* (Sheldrick, 2008 ▶); molecular graphics: *DIAMOND* (Brandenburg & Putz, 2005 ▶); software used to prepare material for publication: *WinGX* (Farrugia, 1999 ▶).

## Supplementary Material

Crystal structure: contains datablock(s) global, I. DOI: 10.1107/S1600536812036562/pk2439sup1.cif

Structure factors: contains datablock(s) I. DOI: 10.1107/S1600536812036562/pk2439Isup2.hkl

Supplementary material file. DOI: 10.1107/S1600536812036562/pk2439Isup3.cml

Additional supplementary materials:  crystallographic information; 3D view; checkCIF report

## Figures and Tables

**Table 1:** Hydrogen-bond geometry (Å, °)

*D*—H⋯*A*	*D*—H	H⋯*A*	*D*⋯*A*	*D*—H⋯*A*
N1—H1*A*⋯O3	0.892 (18)	2.019 (19)	2.8721 (14)	159.7 (16)
N1—H1*B*⋯O4^i^	0.836 (19)	2.204 (19)	3.0202 (14)	165.1 (16)
N2—H2*A*⋯O1	0.848 (19)	1.984 (18)	2.6458 (12)	134.1 (16)
N2—H2*A*⋯O3	0.848 (19)	2.496 (18)	3.2058 (12)	141.7 (15)
N3—H3*A*⋯O3^ii^	0.857 (17)	2.153 (17)	2.9652 (12)	158.2 (15)
N3—H3*A*⋯N4	0.857 (17)	2.288 (16)	2.6879 (15)	108.7 (13)
C4—H4⋯O3^ii^	0.93	2.44	3.1947 (14)	138
C11—H11⋯O4^iii^	0.93	2.53	3.3460 (14)	147
C12—H12⋯O2^iv^	0.93	2.5	3.2901 (14)	142
C14—H14⋯O2^ii^	0.93	2.55	3.1840 (14)	126

Symmetry codes: (i) 
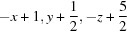
; (ii) 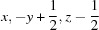; (iii) 
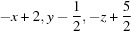
; (iv) 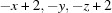.

**Fig. 1.**
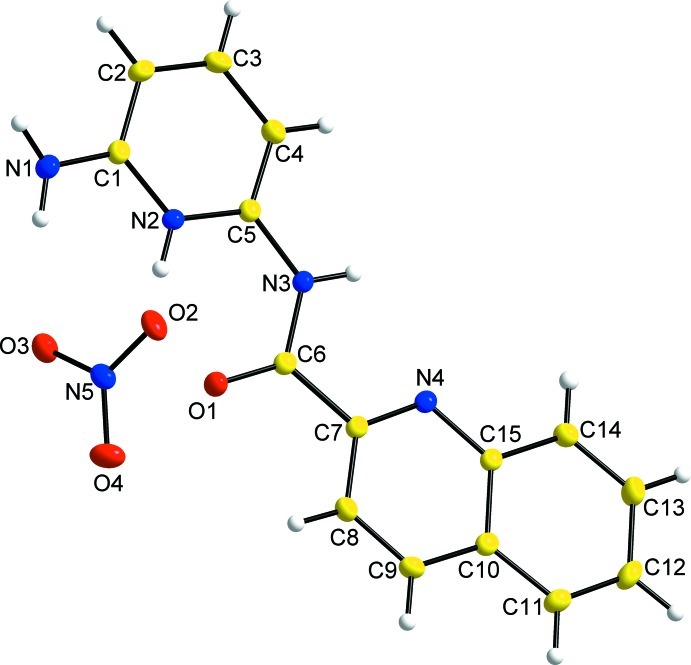
Representation of the title compound, showing displacement ellipsoids (50% probability).

**Fig. 2.**
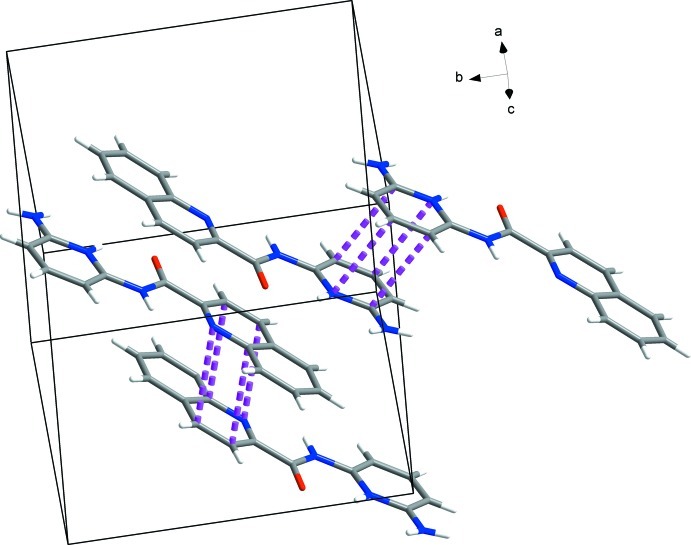
Packing and illustration of π-π stacking in the crystal.

## supplementary crystallographic information

### Comment

The title compound was synthesized as a ligand for potential use in medical and radiopharmaceutical applications. The asymmetric unit in the title compound contains a C_15_H_13_N_4_O cation and a NO_3_^-^ counter ion. A range of N—H···N, N—H···O and C—H···O hydrogen interactions are observed throughout the structure. Further stabilization of the crystal structure is obtained by π-π stacking interactions between inversion-related quinolines and inversion-related pyridines with respective centroid-to-centroid distances of 3.5866 (6) Å and 3.3980 (6) Å (see Fig. 2). For similar structures that form part of our radiopharmaceutical research see: Al-Dajani *et al.* (2010); Jain *et al.* (2004) and Van der Berg *et al.* (2011).

### Experimental

Under oxygen atmosphere: Quinaldic acid (0.8013 g, 4.627 mmol) was added as a solid in one portion to a suspension of 2,6-diaminopyridine (0.5000 g, 4.582 mmol) in pyridine (10 ml) and the mixture was stirred at 40 °C for 40 min. Triphenylphosphite (10 ml) was added dropwise over 10 minutes, after which the temperature was increased to 90–100 °C and stirred for a further 24 h. On cooling the precipitate was filtered, washed with H_2_O (50 ml) and then MeOH (50 ml). The product was dissolved in diluted HNO_3_ and left to stand at room temperature. Yellow crystals were obtained after five days.

### Refinement

The N-bound hydrogen atoms were located in a difference Fourier map and refined freely. The remaining H atoms were placed in geometrically idealized positions at C—H = 0.93 Å, respectively and constrained to ride on their parent atoms, with *U*_iso_(H) = 1.2*U*_eq_(C).

### Crystal data

**Table:**

C_15_H_13_N_4_O^+^·NO_3_^−^	*F*(000) = 680
*M**_r_* = 327.30	*D*_x_ = 1.523 Mg m^−^^3^
Monoclinic, *P*2_1_/*c*	Mo *K*α radiation, λ = 0.71073 Å
Hall symbol: -P 2ybc	Cell parameters from 9936 reflections
*a* = 8.183 (2) Å	θ = 2.2–28.3°
*b* = 11.768 (3) Å	µ = 0.12 mm^−^^1^
*c* = 14.979 (4) Å	*T* = 100 K
β = 98.37 (1)°	Cuboid, yellow
*V* = 1427.1 (6) Å^3^	0.49 × 0.41 × 0.31 mm
*Z* = 4	

### Data collection

**Table:**

Bruker X8 APEXII KappaCCD diffractometer	3555 independent reflections
Radiation source: sealed tube	3180 reflections with *I* > 2σ(*I*)
Graphite monochromator	*R*_int_ = 0.023
φ and ω scans	θ_max_ = 28.4°, θ_min_ = 2.2°
Absorption correction: multi-scan (*SADABS*; Bruker, 2008)	*h* = −10→10
*T*_min_ = 0.990, *T*_max_ = 0.994	*k* = −15→15
26710 measured reflections	*l* = −19→19

### Refinement

**Table:**

Refinement on *F*^2^	Primary atom site location: structure-invariant direct methods
Least-squares matrix: full	Secondary atom site location: difference Fourier map
*R*[*F*^2^ > 2σ(*F*^2^)] = 0.035	Hydrogen site location: inferred from neighbouring sites
*wR*(*F*^2^) = 0.1	H atoms treated by a mixture of independent and constrained refinement
*S* = 1.04	*w* = 1/[σ^2^(*F*_o_^2^) + (0.0537*P*)^2^ + 0.6672*P*] where *P* = (*F*_o_^2^ + 2*F*_c_^2^)/3
3555 reflections	(Δ/σ)_max_ = 0.001
233 parameters	Δρ_max_ = 0.42 e Å^−^^3^
0 restraints	Δρ_min_ = −0.25 e Å^−^^3^

### Special details

**Table:**

Experimental. The intensity data were collected on a Bruker X8 ApexII 4 K Kappa CCD diffractometer using an exposure time of 30 s/frame. A total of 1759 frames were collected with a frame width of 0.5° covering up to θ = 28.28° with 100.00% completeness accomplished.
Geometry. All s.u.'s (except the s.u. in the dihedral angle between two l.s. planes) are estimated using the full covariance matrix. The cell s.u.'s are taken into account individually in the estimation of s.u.'s in distances, angles and torsion angles; correlations between s.u.'s in cell parameters are only used when they are defined by crystal symmetry. An approximate (isotropic) treatment of cell s.u.'s is used for estimating s.u.'s involving l.s. planes.
Refinement. Refinement of *F*^2^ against ALL reflections. The weighted *R*-factor *wR* and goodness of fit *S* are based on *F*^2^, conventional *R*-factors *R* are based on *F*, with *F* set to zero for negative *F*^2^. The threshold expression of *F*^2^ > 2σ(*F*^2^) is used only for calculating *R*-factors(gt) *etc*. and is not relevant to the choice of reflections for refinement. *R*-factors based on *F*^2^ are statistically about twice as large as those based on *F*, and *R*- factors based on ALL data will be even larger.

### Fractional atomic coordinates and isotropic or equivalent isotropic displacement parameters (Å^2^)

**Table:**

	*x*	*y*	*z*	*U*_iso_*/*U*_eq_	
C5	0.44014 (13)	0.29849 (9)	1.00107 (7)	0.0136 (2)	
C4	0.36313 (14)	0.35271 (9)	0.92503 (7)	0.0165 (2)	
H4	0.3674	0.323	0.8679	0.02*	
C3	0.27835 (14)	0.45365 (10)	0.93606 (8)	0.0190 (2)	
H3	0.2243	0.4909	0.8854	0.023*	
C2	0.27319 (14)	0.49912 (10)	1.02018 (8)	0.0191 (2)	
H2	0.2165	0.5665	1.0263	0.023*	
C1	0.35435 (13)	0.44278 (9)	1.09694 (8)	0.0161 (2)	
C6	0.61016 (12)	0.13855 (9)	1.06647 (7)	0.0130 (2)	
C7	0.70961 (12)	0.03990 (9)	1.04078 (7)	0.0128 (2)	
C8	0.80954 (13)	−0.01866 (9)	1.11085 (7)	0.0148 (2)	
H8	0.812	0.0028	1.1708	0.018*	
C9	0.90254 (13)	−0.10779 (9)	1.08810 (7)	0.0156 (2)	
H9	0.9709	−0.1475	1.1325	0.019*	
C10	0.89350 (13)	−0.13878 (9)	0.99620 (7)	0.0143 (2)	
C11	0.98613 (14)	−0.23014 (10)	0.96740 (8)	0.0182 (2)	
H11	1.0548	−0.2727	1.0098	0.022*	
C12	0.97515 (14)	−0.25610 (10)	0.87763 (8)	0.0208 (2)	
H12	1.0371	−0.3157	0.8593	0.025*	
C13	0.86987 (14)	−0.19264 (10)	0.81263 (8)	0.0196 (2)	
H13	0.8633	−0.2111	0.7518	0.024*	
C14	0.77756 (14)	−0.10438 (9)	0.83805 (7)	0.0165 (2)	
H14	0.7078	−0.064	0.7947	0.02*	
C15	0.78850 (13)	−0.07462 (9)	0.93054 (7)	0.0133 (2)	
N1	0.36061 (13)	0.48153 (9)	1.18100 (7)	0.0199 (2)	
N2	0.43161 (11)	0.34266 (8)	1.08430 (6)	0.01391 (19)	
N3	0.53164 (11)	0.20051 (8)	0.99536 (6)	0.01377 (19)	
N4	0.69803 (11)	0.01543 (7)	0.95409 (6)	0.01317 (18)	
N5	0.68666 (12)	0.29972 (8)	1.29263 (6)	0.01620 (19)	
O1	0.60217 (10)	0.16082 (7)	1.14574 (5)	0.01639 (17)	
O2	0.75498 (11)	0.36208 (7)	1.24246 (6)	0.0224 (2)	
O3	0.53418 (10)	0.31398 (7)	1.29794 (5)	0.01974 (18)	
O4	0.76463 (11)	0.22297 (7)	1.33845 (6)	0.0228 (2)	
H1B	0.326 (2)	0.5477 (16)	1.1861 (12)	0.033 (4)*	
H1A	0.423 (2)	0.4444 (15)	1.2254 (12)	0.031 (4)*	
H2A	0.481 (2)	0.3072 (15)	1.1296 (12)	0.032 (4)*	
H3A	0.5452 (19)	0.1790 (14)	0.9423 (11)	0.026 (4)*	

### Atomic displacement parameters (Å^2^)

**Table:**

	*U*^11^	*U*^22^	*U*^33^	*U*^12^	*U*^13^	*U*^23^
C5	0.0144 (4)	0.0117 (5)	0.0151 (5)	−0.0008 (4)	0.0036 (4)	−0.0006 (4)
C4	0.0196 (5)	0.0159 (5)	0.0139 (5)	0.0001 (4)	0.0027 (4)	0.0000 (4)
C3	0.0215 (5)	0.0168 (5)	0.0182 (5)	0.0031 (4)	0.0017 (4)	0.0045 (4)
C2	0.0230 (5)	0.0134 (5)	0.0212 (6)	0.0051 (4)	0.0045 (4)	0.0017 (4)
C1	0.0182 (5)	0.0131 (5)	0.0178 (5)	0.0006 (4)	0.0060 (4)	−0.0002 (4)
C6	0.0127 (4)	0.0112 (5)	0.0152 (5)	−0.0015 (4)	0.0018 (4)	−0.0011 (4)
C7	0.0125 (5)	0.0111 (4)	0.0151 (5)	−0.0010 (3)	0.0027 (4)	−0.0007 (4)
C8	0.0152 (5)	0.0165 (5)	0.0129 (5)	−0.0006 (4)	0.0025 (4)	−0.0002 (4)
C9	0.0141 (5)	0.0165 (5)	0.0158 (5)	0.0010 (4)	0.0006 (4)	0.0031 (4)
C10	0.0125 (5)	0.0130 (5)	0.0177 (5)	−0.0005 (4)	0.0034 (4)	0.0004 (4)
C11	0.0164 (5)	0.0160 (5)	0.0219 (6)	0.0037 (4)	0.0018 (4)	0.0007 (4)
C12	0.0198 (5)	0.0165 (5)	0.0268 (6)	0.0041 (4)	0.0062 (4)	−0.0045 (4)
C13	0.0223 (5)	0.0198 (5)	0.0173 (5)	0.0010 (4)	0.0049 (4)	−0.0043 (4)
C14	0.0191 (5)	0.0153 (5)	0.0150 (5)	0.0006 (4)	0.0018 (4)	−0.0003 (4)
C15	0.0130 (4)	0.0115 (5)	0.0156 (5)	−0.0008 (3)	0.0035 (4)	−0.0001 (4)
N1	0.0288 (5)	0.0151 (5)	0.0166 (5)	0.0058 (4)	0.0059 (4)	−0.0011 (4)
N2	0.0171 (4)	0.0119 (4)	0.0129 (4)	0.0021 (3)	0.0027 (3)	0.0005 (3)
N3	0.0175 (4)	0.0120 (4)	0.0122 (4)	0.0018 (3)	0.0033 (3)	−0.0009 (3)
N4	0.0142 (4)	0.0111 (4)	0.0144 (4)	−0.0003 (3)	0.0026 (3)	−0.0008 (3)
N5	0.0221 (5)	0.0142 (4)	0.0121 (4)	−0.0034 (3)	0.0016 (3)	−0.0018 (3)
O1	0.0202 (4)	0.0155 (4)	0.0133 (4)	0.0019 (3)	0.0020 (3)	−0.0023 (3)
O2	0.0317 (5)	0.0184 (4)	0.0195 (4)	−0.0055 (3)	0.0111 (3)	0.0005 (3)
O3	0.0196 (4)	0.0234 (4)	0.0163 (4)	−0.0020 (3)	0.0031 (3)	−0.0002 (3)
O4	0.0269 (4)	0.0182 (4)	0.0206 (4)	−0.0006 (3)	−0.0052 (3)	0.0025 (3)

### Geometric parameters (Å, º)

**Table:**

C5—N2	1.3618 (14)	C9—H9	0.93
C5—C4	1.3758 (15)	C10—C11	1.4183 (15)
C5—N3	1.3842 (13)	C10—C15	1.4246 (15)
C4—C3	1.3974 (15)	C11—C12	1.3690 (17)
C4—H4	0.93	C11—H11	0.93
C3—C2	1.3753 (16)	C12—C13	1.4156 (17)
C3—H3	0.93	C12—H12	0.93
C2—C1	1.4073 (16)	C13—C14	1.3704 (15)
C2—H2	0.93	C13—H13	0.93
C1—N1	1.3332 (15)	C14—C15	1.4191 (15)
C1—N2	1.3633 (14)	C14—H14	0.93
C6—O1	1.2270 (13)	C15—N4	1.3680 (13)
C6—N3	1.3711 (14)	N1—H1B	0.836 (19)
C6—C7	1.4997 (14)	N1—H1A	0.892 (18)
C7—N4	1.3200 (13)	N2—H2A	0.848 (19)
C7—C8	1.4125 (14)	N3—H3A	0.857 (17)
C8—C9	1.3679 (15)	N5—O2	1.2402 (12)
C8—H8	0.93	N5—O4	1.2516 (13)
C9—C10	1.4155 (15)	N5—O3	1.2729 (13)
			
N2—C5—C4	120.18 (10)	C11—C10—C15	119.15 (10)
N2—C5—N3	118.34 (9)	C12—C11—C10	120.42 (10)
C4—C5—N3	121.46 (10)	C12—C11—H11	119.8
C5—C4—C3	118.15 (10)	C10—C11—H11	119.8
C5—C4—H4	120.9	C11—C12—C13	120.28 (10)
C3—C4—H4	120.9	C11—C12—H12	119.9
C2—C3—C4	121.40 (10)	C13—C12—H12	119.9
C2—C3—H3	119.3	C14—C13—C12	120.90 (10)
C4—C3—H3	119.3	C14—C13—H13	119.5
C3—C2—C1	119.44 (10)	C12—C13—H13	119.5
C3—C2—H2	120.3	C13—C14—C15	119.95 (10)
C1—C2—H2	120.3	C13—C14—H14	120
N1—C1—N2	118.16 (10)	C15—C14—H14	120
N1—C1—C2	124.00 (10)	N4—C15—C14	118.90 (10)
N2—C1—C2	117.84 (10)	N4—C15—C10	121.82 (10)
O1—C6—N3	123.59 (10)	C14—C15—C10	119.28 (10)
O1—C6—C7	121.40 (9)	C1—N1—H1B	116.0 (12)
N3—C6—C7	115.01 (9)	C1—N1—H1A	118.4 (11)
N4—C7—C8	125.13 (10)	H1B—N1—H1A	123.6 (16)
N4—C7—C6	117.22 (9)	C5—N2—C1	122.93 (10)
C8—C7—C6	117.65 (9)	C5—N2—H2A	117.6 (12)
C9—C8—C7	118.16 (10)	C1—N2—H2A	119.4 (12)
C9—C8—H8	120.9	C6—N3—C5	126.27 (9)
C7—C8—H8	120.9	C6—N3—H3A	117.0 (11)
C8—C9—C10	119.18 (10)	C5—N3—H3A	116.6 (11)
C8—C9—H9	120.4	C7—N4—C15	117.29 (9)
C10—C9—H9	120.4	O2—N5—O4	121.37 (10)
C9—C10—C11	122.44 (10)	O2—N5—O3	119.49 (9)
C9—C10—C15	118.40 (9)	O4—N5—O3	119.14 (9)

### Hydrogen-bond geometry (Å, º)

**Table:**

*D*—H···*A*	*D*—H	H···*A*	*D*···*A*	*D*—H···*A*
N1—H1*A*···O3	0.892 (18)	2.019 (19)	2.8721 (14)	159.7 (16)
N1—H1*B*···O4^i^	0.836 (19)	2.204 (19)	3.0202 (14)	165.1 (16)
N2—H2*A*···O1	0.848 (19)	1.984 (18)	2.6458 (12)	134.1 (16)
N2—H2*A*···O3	0.848 (19)	2.496 (18)	3.2058 (12)	141.7 (15)
N3—H3*A*···O3^ii^	0.857 (17)	2.153 (17)	2.9652 (12)	158.2 (15)
N3—H3*A*···N4	0.857 (17)	2.288 (16)	2.6879 (15)	108.7 (13)
C4—H4···O3^ii^	0.93	2.44	3.1947 (14)	138
C11—H11···O4^iii^	0.93	2.53	3.3460 (14)	147
C12—H12···O2^iv^	0.93	2.5	3.2901 (14)	142
C14—H14···O2^ii^	0.93	2.55	3.1840 (14)	126

Symmetry codes: (i) −*x*+1, *y*+1/2, −*z*+5/2; (ii) *x*, −*y*+1/2, *z*−1/2; (iii) −*x*+2, *y*−1/2, −*z*+5/2; (iv) −*x*+2, −*y*, −*z*+2.

## References

[bb1] Al-Dajani, M. T. M., Mohamed, N., Wahab, H. A., Yeap, C. S. & Fun, H.-K. (2010). *Acta Cryst.* E**66**, o2150. 10.1107/S1600536810029624 PMC3007299 21588436

[bb2] Brandenburg, K. & Putz, H. (2005). *DIAMOND* Crystal Impact GbR, Bonn, Germany.

[bb3] Bruker (2008). *SAINT-Plus* and *SADABS* Bruker AXS Inc., Madison, Wisconsin, USA.

[bb4] Bruker (2011). *APEX2* Bruker AXS Inc., Madison, Wisconsin, USA.

[bb5] Farrugia, L. J. (1999). *J. Appl. Cryst.* **32**, 837–838.

[bb6] Jain, S. L., Bhattacharyya, P., Milton, H. L., Slawin, A. M. Z., Crayston, J. A. & Woollins, J. D. (2004). *Dalton Trans.* pp. 862–871. 10.1039/b316519a 15252470

[bb7] Sheldrick, G. M. (2008). *Acta Cryst.* A**64**, 112–122. 10.1107/S0108767307043930 18156677

[bb8] Van der Berg, P. C. W., Visser, H. G. & Roodt, A. (2011). *Acta Cryst.* E**67**, o3130. 10.1107/S160053681104414X PMC3247514 22220132

